# Watermelon and dietary advice compared to dietary advice alone following hospitalization for hyperemesis gravidarum: a randomized controlled trial

**DOI:** 10.1186/s12884-023-05771-7

**Published:** 2023-06-17

**Authors:** Peng Chiong Tan, Gayaithiri Ramasandran, Neha Sethi, Nuguelis Razali, Mukhri Hamdan, Maherah Kamarudin

**Affiliations:** Department of Obstetrics and Gynecology, Faculty of Medicine, Universiti Malaya, Jalan Profesor Diraja Ungku Aziz, 50603 Kuala Lumpur, Malaysia

**Keywords:** Hyperemesis gravidarum, Nausea and vomiting of pregnancy, Watermelon, Appetite, Maternal satisfaction, Dietary advice

## Abstract

**Background:**

Hyperemesis gravidarum (HG) affects about 2% of pregnancies and is at the severe end of the spectrum of nausea and vomiting of pregnancy. HG causes severe maternal distress and results in adverse pregnancy outcomes long after the condition may have dissipated. Although dietary advice is a common tool in management, trial evidence to base the advice on is lacking.

**Methods:**

A randomized trial was conducted in a university hospital from May 2019 to December 2020. 128 women at their discharge following hospitalization for HG were randomized: 64 to watermelon and 64 to control arm. Women were randomized to consume watermelon and to heed the advice leaflet or to heed the dietary advice leaflet alone. A personal weighing scale and a weighing protocol were provided to all participants to take home. Primary outcomes were bodyweight change at the end of week 1 and week 2 compared to hospital discharge.

**Results:**

Weight change (kg) at end of week 1, median[interquartile range] -0.05[-0.775 to + 0.50] vs. -0.5[-1.4 to + 0.1] *P* = 0.014 and to the end of week 2, + 0.25[-0.65 to + 0.975] vs. -0.5[-1.3 to + 0.2] *P* = 0.001 for watermelon and control arms respectively. After two weeks, HG symptoms assessed by PUQE-24 (Pregnancy-Unique Quantification of Emesis and Nausea over 24 h), appetite assessed by SNAQ (Simplified Nutritional Appetite Questionnaire), wellbeing and satisfaction with allocated intervention NRS (0–10 numerical rating scale) scores, and recommendation of allocated intervention to a friend rate were all significantly better in the watermelon arm. However, rehospitalization for HG and antiemetic usage were not significantly different.

**Conclusion:**

Adding watermelon to the diet after hospital discharge for HG improves bodyweight, HG symptoms, appetite, wellbeing and satisfaction.

**Trial registration:**

This study was registered with the center’s Medical Ethics Committee (on 21/05/2019; reference number 2019327–7262) and the ISRCTN on 24/05/2019 with trial identification number: ISRCTN96125404. First participant was recruited on 31/05/ 2019.

**Supplementary Information:**

The online version contains supplementary material available at 10.1186/s12884-023-05771-7.

## Background

Hyperemesis gravidarum (HG) affects about 0.3–3.6% of pregnant women [1]. The milder nausea and vomiting in pregnancy (NVP) is experienced by up to 90% of pregnancies [2]. By 8 weeks of pregnancy, 57.3% reports nausea and 26.6% nausea with vomiting [3]. HG is defined as severe nausea and/or vomiting starting before 16 weeks with inability to eat and/or drink normally and daily living activities are strongly limited [4]. Weight loss and dehydration are common clinical features in HG. Many women describe HG as one of their worst life experiences [5]. Anemia, pre-eclampsia, eclampsia, venous thromboembolism, preterm and very preterm birth, cesarean birth, low birth-weight or small for gestational age and neonatal intensive care are associated with HG [6].

Nutrition support, consequences of malnutrition and dehydration and the role of oral supplements, fortifying food and dietary measure to achieve nutritional requirements rank amongst top 10 HG research priorities identified by a large study of stakeholders representing patients, carers and multidisciplinary professionals [7]. A 2020 review on nutritional intake in HG finds only four papers published over a 30-year span with data from 314 women, identifying a paucity of data [8].

In women hospitalized for HG, bitter was most likely (32%) and sweet taste least likely (5%) to provoke nausea or vomiting on taste strip testing. On questionnaire response, crunchy sweet uncooked food (apple or watermelon) was best tolerated [9]. In a food tasting trial in women hospitalized for HG, apple and watermelon top the agreeability score and have the lowest nausea and emesis response rates amongst the food items consumed [10]. The watermelon have excellent hydration potential; its water content which is in excess of 90% is one of the highest amongst fruits and vegetables [11]. Watermelon is considered to have a low glycemic index amongst local Malaysian fruits [12].

We postulate that adding fresh watermelon to the diet of women after their hospitalization for HG as it is agreeable and tolerated, will positively impact on bodyweight driven by better hydration from tolerated intake. The confidence building from watermelon “staying down” after being eaten may encourage the consumption of other food and drink and hasten recovery from HG. We performed a randomized controlled trial to test the hypothesis.

## Methods

This is a randomized controlled trial comparing watermelon and dietary advice leaflet to dietary advice leaflet only (control) at hospital discharge for HG. The trial was approved by our center’s Medical Ethics Committee (approval on 21/05/2019; reference number 2019327–7262) and registered in the International Standard Randomized Controlled Trials Number registry on 24/05/2019; reference number ISRCTN96125404 (https://doi.org/10.1186/ISRCTN96125404) prior to trial enrolment. Informed consent was taken from all participants and research has been performed in accordance with the Declaration of Helsinki. The first participant was recruited on 31 May, 2019 and the last on 18 December, 2020.

Our HG cases were characterized ‘intractable nausea and vomiting of pregnancy with dehydration and starvation clinically judged to require hospitalization for intravenous rehydration and antiemetic drug administration’ [13]. We had shown that HG cases admitted to our centre have ‘comparable metabolic and biochemical characteristics to previous studies of hyperemesis gravidarum’ [14]. Women admitted for HG in our centre typically ‘received our standard inpatient care for HG, which comprised intravenous rehydration with normal saline solution (with potassium chloride added if required for hypokalaemia), intravenous anti-emetic drug (first-line 10 mg metoclopramide 8-hourly and supplementation with oral thiamine’ and ‘were encouraged to resume oral intake of both fluid and solid as soon as, as much as and as often as could be tolerated’ [15].

Women were assessed for eligibility by scrutinizing their medical records during their inpatient management of HG in the gynecology ward, University Malaya Medical Center, Kuala Lumpur, Malaysia usually on the day of discharge. Inclusion criteria were a diagnosis of HG, gestation age less than 16 weeks [4], age ≥ 18 years and first hospitalization for HG in the current pregnancy. Exclusion criteria were confirmed non-viable pregnancy, allergy or intolerance to watermelon. aversion to watermelon and multiple gestations. Eligible women were approached, given the Patient Information Sheet and their oral queries were addressed by the recruiting investigator (co-author GR). Written informed consent was obtained.

Pregnancy-Unique Quantification of Emesis (PUQE-24) score [16], Simplified Nutritional Appetite Questionnaire (SNAQ) score [17], and an 11-point NRS (0–10 numerical rating scale, high score greater wellbeing) were obtained at ward discharge. Participants’ characteristics were transcribed onto the Case Report Form.

We avoided the mention of watermelon as much as possible during the recruitment process to minimize confounding arising from women in the control arm being motivated to consume watermelon. The study title in the Patient Information Sheet was “The effect of standard dietary advice in hyperemesis gravidarum patients: a prospective trial”; this approach was approved by the Ethics board.

The randomization sequence was generated in random blocks of 4 or 8 using random number generator at random.org by investigator (co-author PCT) who was not involved in enrolment. Numbered sealed opaque envelopes were prepared. The lowest numbered envelope available was assigned to the newest recruit. Randomization was by opening the sealed envelope just prior to discharge.

Participants randomized to watermelon and dietary advice leaflet were supplied with two fresh red-fleshed watermelon (approximately 4 kg weight) to take home (Supplementary material S1) [18] in addition to the dietary advice leaflet which they were advised to read and heed. The watermelons were sourced from a local supermarket at approximate cost of USD 3 per fruit. Written instruction was given on fruit handling, storage and hygiene (Supplementary material S2). These participants were instructed to consume 1/8 of the whole fruit flesh daily, in further divided portions for the following two weeks and to read and heed the dietary advice leaflet. The advice leaflet was sourced online from a UK NHS university hospital trust website and freely available for patient information. Participants randomized to advice leaflet were provided with an identical advice leaflet to read and heed.

Identical, commercially procured ordinary electronic weighing scales (providing weight measure in 0.1 kg increments) were provided free of charge to every participant for their exclusive use within the trial. The participants used their allocated personal weighing scale for the predischarge, week 1 and week 2 weighings. A written standard operating procedure provided instructions on the timing, clothing worn and bladder emptying requirements for the weighing (Supplementary material S3). At each weighing, three weights were obtained with the middle value taken if all three were discrepant and the concordant value taken if at least two of the three readings were identical,

Participants were not blinded to their allocated intervention as the nature of the interventions were obvious. However, the mention of watermelon was avoided prior to randomization and subsequently in interaction with the controls.

Primary outcomes were change in bodyweight in 1st week and through into the 2nd week after hospital discharge compared to bodyweight at discharge, evaluated across trial arms. Main secondary outcomes were HG symptoms assessed with PUQE-24 [16], appetite evaluated by SNAQ [17] at 1 week and 2 weeks after discharge. Wellbeing NRS score and date of last use of oral antiemetics (if stopped) were also obtained at the end of 1 week and 2 weeks after discharge. At the end of 2 weeks, participants were asked their satisfaction with the allocated intervention, if they would recommend their intervention to a friend or family member and whether they were rehospitalized for HG. The above assessments were through telephone interview.

For sample size calculation, we postulate a 0.5 kg difference in weight change by the end of week 1 across trial arms with a standard deviation (SD) of 1 kg in the weight change distribution for both arms. Applying α of 0.05, power of 80%, and 1 to 1 randomization ratio using Student t-test for analysis, 64 participants are required in each arm (*N* = 128). At the end of 2 weeks after discharge, we postulated a weight change difference of 0.75 kg difference across trial arms with SD of 1.5 kg in both arms. Applying α of 0.05, power of 80%, and 1 to 1 randomization ratio using t-test for analysis, 64 participants are also required in each arm (*N* = 128). We planned to recruit 128 participants.

Data were entered into SPSS (Version 23, IBM, SPSS Statistics). The 1-sample Kolmogorov–Smirnov test was used to check for normal distribution of continuous data. Data were expressed as numbers (%), mean ± standard deviation (normally distributed continuous data) or median [interquartile range IQR] (ordinal or non-normally distributed continuous data). The t test was used to compare means where data is normally distributed and the Mann–Whitney U test applied to non-normally distributed or ordinal data. Nominal data sets were analyzed with the Chi-square test. Two-sided P values were reported. *P* < 0.05 is considered significant. Analysis was based on intention-to-treat.

## Results

**Fig. 1:**
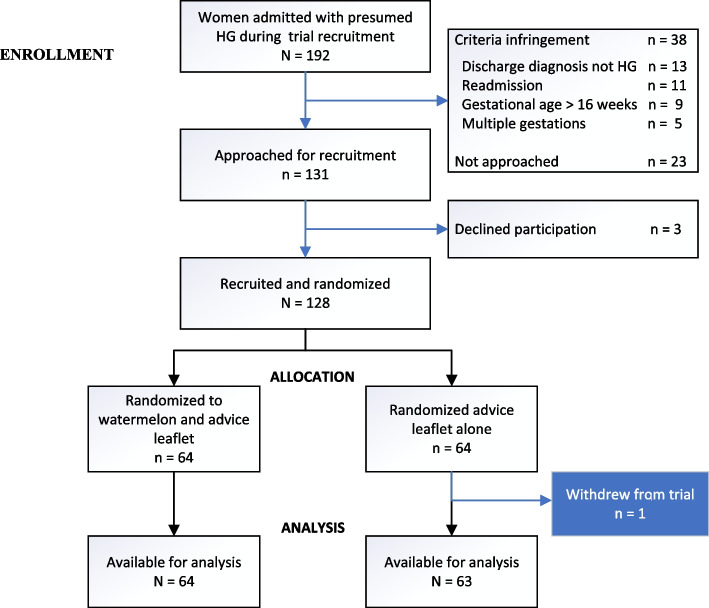
Flow Diagram of a Randomized Controlled Trial Comparing Watermelon And Dietary Advice to Dietary Advice Alone Following Hospitalization for Hyperemesis Gravidarum (HG)

Figure 1 depicts the recruitment flow. Of the 192 ward admissions for presumed HG during trial enrolment, 38 were excluded due to criteria infringement and 23 were not approached. Of the 131 eligible women approached, 3 declined: 128 provided written informed consent to participate. 64 were randomized to each arm. One participant randomized to the control arm withdrew due to bereavement early on in the trial period and did not provide any outcome data. Outcome data from 127 participants were analyzed. Trial recruitment was stopped on achieving target sample size of 128.

Table 1 shows the participants’ characteristics dichotomized according to their allocated intervention. Characteristics were not significantly different across trial arms: specifically, for bodyweight and body mass index, scores for PUQE-24, SNAQ and wellbeing, hospital stay duration and antiemetic prescribed on recruitment at their hospital discharge.

**Table 1:** Characteristics of participants randomized to watermelon and dietary advice versus dietary advice only following hospitalization for hyperemesis gravidarum

Characteristics	Watermelon and dietary advice *n* = 64	Dietary advice only *n* = 64	*P* value
Age (years)	30.8 ± 4.4	30.8 ± 4.8	0.98
Gestational age (weeks)	9.2 ± 2.1	9.5 ± 2.1	0.44
Parity			0.88
0	30 (46.9%)	27 (42.2%)	
1	13 (20.3%)	19 (29.7%)	
2	13 (20.3%)	11 (17.2%)	
≥ 3	8 (12.5%)	7 (10.9%)	
Previous miscarriage	18 (28.1%)	14 (21.9%)	0.41
*Ethnicity*			0.32
Malay	52 (81.3%)	47 (73.4%)	
Indian	10 (15.6%)	11 (17.2%)	
Chinese	0 (0%)	3 (4.7%)	
Other	2 (3.1%)	3 (4.7%)	
*Education level*			0.25
Up to secondary	18 (29.1%)	12 (18.8%)	
Diploma	29 (45.3%)	27 (42.2%)	
Degree and beyond	17 (26.6%)	25 (39.1%)	
*Occupation*			0.10
Paid employment	45 (70.3%)	53 (82.8%)	
Homemakers and students	19 (29.7%)	11 (17.2%)	
*At hospital discharge*			
Body mass index (kg/m^2^)	24.6 ± 5.3	24.4 ± 4.2	0.94
Weight (kilogram)	59.8 ± 13.8	61.0 ± 11.2	0.54
Hospital stay (days)	1 [1–2]	1.5 [1–2]	0.68
PUQE-24^a^ score [3 to 15]	9 [6–9]	8 [6–9]	0.86
PUQE-24 symptom category^a^			> 0.99
Mild symptoms	17 (28.6%)	17 (28.6%)	
Moderate symptoms	44 (68.6%)	44 (68.6%)	
Severe symptoms	3 (4.7%)	3 (4.7%)	
SNAQ^b^ score (4 to 16)	11 [10–13]	11 [10–13]	0.89
Significant risk of weight loss^b^	56 (87.5%)	54 (84.4%)	0.61
Wellbeing^c^ score (0 to 10)	6 [5–7]	6 [5–7]	0.38
Antiemetic prescribed			0.17
Oral metoclopramide only	63 (98.4%)	60 (93.8%)	
Others^d^	1 (1.6%)	4 (6.3%)	

Data expressed as mean ± standard deviation for continuous data, number (%) for categoric data and median {interquartile range] for ordinal or non-normally distributed data. Analyses performed using Student t test for continuous data, Chi-square test for categoric data or Mann Whitney u test for ordinal data or non-normally distributed data. 2-sided analyses *P* < 0.05 taken as significant for all variables

^a^PUQE-24 (Pregnancy-Unique Quantification of Emesis and Nausea over 24 h) scoring system for nausea and vomiting of pregnancy. J Obstet Gynaecol Can. 2009 Sep;31(9):803–807 scored from 3 to 15. Symptoms graded as mild ≤ 6 score, moderate 7–12 score and severe ≥ 13 score

^b^Simplified Nutritional Appetite Questionnaire (SNAQ). Am J Clin Nutr. 2005 Nov;82(5):1074–81 scored from 4 to 16. Significant risk of weight loss ≤ 14 score

^c^Wellbeing score from 0 to 10 by participants (higher score greater wellbeing)

^d^Ondansetron 1 (watermelon arm); metoclopramide, domperidone and Veloxin 1, ondansetron 1 and Veloxin 2 (control arm) all antiemetics to be taken orally

Table 2 reports the primary outcomes. Weight change (kg) at end of week 1, median [interquartile range] -0.05 [-0.775 to + 0.50] vs. -0.5 [-1.4 to + 0.1] *P* = 0.014 and to the end of week 2, + 0.25 [-0.65 to + 0.975] vs. -0.5 [-1.3 to + 0.2] *P* = 0.001 for watermelon and control arms respectively. On the post hoc categoric weight change metric of “lost weight”, in week 1 the rate was 32/64 (50.0%) vs. 45/63 (71.4%) RR [Relative Risk] (95% CI [confidence interval]) 0.70 (0.52–0.94) NNT_b_ [number needed to treat to benefit] (95% CI) 4.7 (2.6–20.6) *P* = 0.013 and through to week 2, 26/64 (40.6%) vs. 39/63 (61.9%) RR (95% CI) 0.66 (0.48–0.94) NNT_b_ (95% CI) 4.7 (2.6–23.3) *P* = 0.016 for watermelon and control arms respectively. These bodyweight metrics favored the watermelon arm.

**Table 2:** Primary outcome of weight change and weight related outcome after randomization to watermelon and dietary advice versus dietary advice only following hospitalization for hyperemesis gravidarum

Outcomes	Watermelon and dietary advice *n* = 64	Dietary advice only *n* = 63	RR (95% CI)	NNTb (95% CI)	*P* value
Week 1^a^					
Weight change (kg)	-0.05 [-0.775 to + 0.50]	-0.5 [-1.4 to + 0.1]			0.014
Lost weight	32 (50%)	45 (71.4%)	0.70 (0.52–0.94)	4.7 (2.6–20.6)	0.013
Week 2^b^					
Weight change (kg)	+ 0.25 [-0.65 to + 0.975]	-0.5 [-1.3 to + 0.2]			0.001
Lost weight	26 (40.6%)	39 (61.9%)	0.66 (0.48–0.94)	4.7 (2.6–23.3)	0.016

Data expressed as median {interquartile range] for non-normally distributed continuous data and number (%) for categoric data. Analyses performed using Mann Whitney u test for non-normally distributed continuous data or Chi-square test for categoric data. 2-sided analyses *P* < 0.05 taken as significant for all variables

^a^From discharge to the end of week 1

^b^From discharge to the end of week 2

Table 3 shows the result for main secondary and other outcomes. PUQE-24, SNAQ and wellbeing scores although improved at the end of week 1 in the watermelon arm, the difference did not achieve statistical significant. By the end of week 2, PUQE -24 score median [interquartile range] was 5[3–6] vs. 6[4–7] *P* = 0.042, those categorized as mildly symptomatic 50/64(78.1%) vs. 38/63(60.3%) RR (95% CI) 1.30 (1.02–1.64) NNT_b_ (95% CI) 5.6 (3.0–48.5) *p* = 0.03, SNAQ score was 15 [14–16] vs. 14 [13–16] *P* = 0.015 and those categorized as at significant risk of weight loss 17/64(26.6%) vs. 30/63(47.6%) RR (95% CI) 0.51 (0.32–0.81) NNT_b_ (95% CI) 4.7 (2.7–21.4) *P* = 0.003 for watermelon and control arms respectively, were statistically significantly different across trial arms and all favored the watermelon arm. Wellbeing score, satisfaction with intervention score and recommendation of intervention to a friend rate were all also significantly higher in the watermelon arm. However, rehospitalization for HG rate 6/64 (9.4%) vs. 6/63 (9.5%) RR 95% CI 0.99 (0.34–2.89) *P* = 0.98 and antiemetic usage metrics were not different. No participant suffered major harms of food poisoning or Wernicke’s encephalopathy within the trial follow up.

**Table 3:** Secondary outcomes after randomization to watermelon and dietary advice versus dietary advice only following hospitalization for hyperemesis gravidarum

Outcomes	Watermelon and dietary advice *n* = 64	Dietary advice only *n* = 63	RR (95% CI)	NNTb (95% CI)	*P* value
Week 1					
PUQE-24^a^ score (3 to 15)	6.5 [5–8]	7 [5–8]			0.44
PUQE-24 symptom category^a^					0.34
Mild symptoms	32 (50.0%)	26 (41.3%)	1.23 (0.84–1.81)^b^		0.29^b^
Moderate symptoms	31 (48.4%)	37 (58.7%)			
Severe symptoms	1 (4.7%)	0 (4.7%)			
SNAQ^c^ score (4 to 16)	14 [11–15]	13 [11–14]			0.20
Significant risk of weight loss	44 (68.8%)	50 (79.8%)	0.87 (0.70–1.07)		0.17
Wellbeing^d^ score (0 to 10)	7 [6–7.75]	7 [6–7]			0.83
Week 2				-	
PUQE-24^a^ score (3 to 15)	5 [3–6]	6 [4–7]			0.042
PUQE-24 symptom category^a^					0.086
Mild symptoms	50 (78.1%)	38 (60.3%)	1.30 (1.02–1.64)^b^	5.6 (3.0–48.5)	0.03^b^
Moderate symptoms	13 (20.3%)	24 (38.1%)			
Severe symptoms	1 (1.6%)	1 (1.6%)			
SNAQ^c^ score (4 to 16)	15 [14–16]	14 [13–16]			0.015
Significant risk of weight loss	17 (26.6%)	30 (47.6%)	0.51 (0.32–0.81)	4.7 (2.7–21.4)	0.003
Wellbeing^d^ score (0 to 10)	8 [7–9]	7 [7–8]			0.015
Satisfaction with intervention^e^	7 [6–8]	6 [5–7]			< 0.001
Recommends intervention	54 (84.4%)	44 (65.1%)	1.30 (1.05–1.60)	5.2 (2.9–21.9)	0.012
Rehospitalisation^f^	6 (9.4%)	6 (9.5%)	0.99 (0.34–2.89)		0.98
Antiemetic stoppage (days)	6 [2–10]	7 [4–13]			0.12
Antiemetic into week 2	19 (29.7)	27 (42.9)	0.59 (0.42–1.61)		0.12
Regular intake of watermelon^g^	54 (84.4%)	g			

Data expressed as median {interquartile range] for ordinal or non-normally distributed continuous data and number (%) for categoric data. Analyses performed using Mann Whitney u test for non-normally distributed data or Chi-square test for categoric data. 2-sided analyses *P* < 0.05 taken as significant for all variables

^a^PUQE-24 (Pregnancy-Unique Quantification of Emesis and Nausea over 24 h) scoring system for nausea and vomiting of pregnancy. J Obstet Gynaecol Can. 2009 Sep;31(9):803–807 scored from 3 to 15. Symptoms graded as mild ≤ 6 score, moderate 7–12 score and severe ≥ 13 score

^b^Analysis of dichotomized mild compared to moderate-severe symptoms

^c^SNAQ Simplified Nutritional Appetite Questionnaire (SNAQ). Am J Clin Nutr. 2005 Nov;82(5):1074–81 scored from 4 to 16. Significant risk of weight loss ≤ 14 score

^d^Wellbeing score from 0 to 10 by participants (higher score greater wellbeing)

^e^Satisfaction score from 0 to 10 by participants (higher score greater satisfaction)

^f^Readmission for inpatient care in the 2 weeks trial period for hyperemesis gravidarum

^g^Directed to participants randomized to watermelon arm only

## Discussion

Weight change metrics over the 2 weeks trial period following hospital discharge for HG were significantly better for the watermelon arm both at the end of the first week and through to the end of the second week as hypothesized. PUQE-24, SNAQ and wellbeing point estimates were also better at end of week 1 in the watermelon arm but these across arm differences only reached significance at the 5% level by the end of week 2. In addition, maternal satisfaction was higher and recommendation of intervention rate to friend or family of allocated intervention was also higher in the watermelon arm. The confluence of these findings points to a consistent positive impact of watermelon. However, on rehospitalization for HG, there was no difference.

By the end of week 2, there was a 0.75 kg difference in weight change across trial arms, equivalent to body weight change of about 1.4%. HG has been defined as protracted NVP with the triad of more than 5% prepregnancy weight loss, dehydration and electrolyte imbalance [19]. In pregnancies affected by HG, inadequate total maternal weight gain and not regaining prepregnancy weight by week 13–18 is associated with small for gestational age [20] and lack of catch-up in gestational weight gain up to the 2nd trimester associated with reduced fetal growth [21].

In overweight and obese adults, daily consumption of watermelon elicited more robust satiety responses, decreased body weight and body mass index [22]. These findings are in contrast to ours on weight change metrics and better appetite with daily watermelon consumption in the very different context of HG rather than for weight control. Dietary compensation is weaker for beverage compared to solid form with total daily energy intake significantly higher with watermelon juice than watermelon fruit; thus watermelon juice may have greater potential for promoting positive energy balance [23] but this premise on watermelon texture is not tested in our HG trial.

Rehospitalization for HG within the next two weeks of the trial period was 9.4%-9.5%; this compared with a 2009 trial report from our center that reports a rehospitalization of 21.1–37.5% in the two weeks after hospital discharge for HG; indicating that the dietary advice leaflet might have a positive effect when compared to historic data [24]. 93.8–98.4% of participants in this trial were discharged with solely oral metoclopramide: intravenous metoclopramide is first line antiemetic in our center for the inpatient treatment of HG [15] with good response [25] and responders were continued with metoclopramide orally after discharge.

A 2021 narrative review on nutritional management of HG finds limited evidence-based research on the effectiveness of dietary approaches [26]. In addition, a 2016 systematic review on treatments for HG and nausea and vomiting in pregnancy [27], a 2017 Cochrane systematic review and meta-analysis on interventions for treating HG [28] and a 2018 network meta-analysis of randomized clinical trials on interventions for treating HG, all do not identify any dietary intervention trial indicating a lack of data to directly compare with our findings of watermelon as a tolerated dietary item.

We designed our trial with an “interventional” control arm of a dietary advice leaflet. Effective treatment of HG requires a combination of medical interventions, lifestyle changes, dietary changes, supportive care, and patient education [26]. However, we are not aware of trial evidence that a dietary advice leaflet is effective in HG, so it might be a sham intervention. As even ‘open-label placebos’ can have a significant overall effect across many scenarios [29] as do sham interventions [30], the dietary advice leaflet was also added to the watermelon arm to permit evaluation of the pure impact of consuming watermelon as an intervention. A 2015 systematic review and meta-analysis on placebo effects concludes that ethical arguments frequently raised against sham-controlled trials were generally not substantiated [30]. A formal dietary advice leaflet was not a component of our routine HG care.

The trial intervention of watermelon consumption and dietary advice leaflet compared to dietary advice leaflet alone resulted in significant clinical improvements: in reducing further weight loss (NNT_b_ 4.7 through both week 1 and week 2), increasing the proportion with none-mild symptoms (PUQE-24 category) by week 2 (NNT_b_ 5.6) and decreasing the proportion at significant risk of weight loss (SNAQ category) by end of week 2 (NNT_b_ 4.7). These clinical gains were underpinned by improvements in the more subjective responses of wellbeing score, satisfaction with intervention score and recommendation of the allocated intervention (NNTb 5.2) by participants in the watermelon arm. The trial findings need corroboration.

As to strengths, the trial is original in the evaluation of a simple diet-based approach to managing HG. The trial is powered to a realistic impact size estimation and the findings are in line with our hypothesis. There was minimal loss of data with only one participant (control arm) who withdrew due to bereavement. Self-reported compliance was good. The trial was designed to minimize confounding from control arm watermelon consumption and from suggestion and placebo effect impacts. The findings are believed to be generalizable to other HG populations as watermelon is a widely available fruit and not a particularly ‘acquired taste’ food item that could case issues with acceptability and compliance.

As to limitations, the trial with a follow up of only 2 weeks, was not informative of a longer-term impact. Outcomes like inability to tolerate oral fluids or food, dehydration, daily functioning and considering terminating a wanted pregnancy as outcomes [31] were not assessed. Nutritional intake was also not assessed in this study. The trial was not powered to evaluate outcomes such as rehospitalization and anti-emetic use.

## Conclusions

In conclusions, two weeks following their discharge from hospitalization for hyperemesis gravidarum, watermelon consumption can help reduce weight loss, lower nausea and vomiting, improve appetite, generate wellbeing and increase patient satisfaction.

## Supplementary Information

**Additional file 1: Supplementary Material S1.** Dietary advice leaflet.

**Additional file 2: Supplementary Material S2.** Standard operating protocol: food preparation and storage.

**Additional file 3: Supplementary Material S3.** Standard operating protocol: body weight measurement.

## Data Availability

The datasets used and/or analyzed during the current study are available from the corresponding author on reasonable request.
