# Supplementary material for: Watermelon and dietary advice compared to dietary advice alone following hospitalization for hyperemesis gravidarum: a randomized controlled trial
Source: BMC Pregnancy Childbirth. 2023 Jun 17;23:450. doi: 10.1186/s12884-023-05771-7 (PMC10276427; doi:10.1186/s12884-023-05771-7)
Supplement: Supplementary file 1 — Additional file 1: Supplementary Material S1. Dietary advice leaflet. [file 12884_2023_5771_MOESM1_ESM.docx]

**HYPEREMESIS GRAVIDARUM**

Hyperemesis gravidarum is a severe form of pregnancy sickness that effects 0.3% to 3.6% of women during early pregnancy.

The persistent vomiting associated with hyperemesis gravidarum can lead to symptoms of dehydration, weight loss, nutritional depletion, tiredness and dizziness. When these symptoms are severe, admission to hospital may be needed for observation, and treatment of dehydration with intravenous fluids. Usually, the hospital admission will only last a few days.

The best advice for anyone suffering from hyperemesis gravidarum is to get plenty of rest and drinks lots of fluids. You will also likely have anti-sickness medication prescribed. The condition usually subsides by week 12 of pregnancy and with early diagnosis and treatment there is no reason why you should not expect a healthy pregnancy.

This information leaflet provides tips and ideas that may help relieve symptoms of nausea and vomiting or help prevent severe dehydration and weight loss. You should try to eat as wide a variety of foods from each food group as possible to avoid missing out on important nutrients for you and your baby. You may need high protein and high calorie foods if you are having trouble eating sufficient amount. The suggestions here are not exhaustive and what works for some may not work for others. Ultimately you should try to consume any foods or drinks that you personally find to be the most appetizing and best tolerated. Remember it is still important to follow current guidelines regarding avoidance of certain food that may contain high level of harmful bacteria or vitamin A e.g. pate, liver, shellfish, soft cheese, undercooked eggs.

**TRY THE FOLLOWING TO HELP RELEIVE NAUSEA & VOMITING**

- Have a small frequent meals and graze on snacks every 2-3 hours rather than trying to have three larger meals daily
- Make the most of times when nausea and vomiting has subsided- you do not have to wait until mealtimes to eat
- Try to eat a small snack one hour before going to sleep at night. This may prevent nausea in the morning when you wake up
- Chew and swallow your foods slowly. Do not rush your meals
- Avoid strong cooking smells. Cold, drier food with less smell may be better tolerated
- If possible, ask someone else to do the cooking or have meals and snacks available that require little preparation
- Drinks most of your fluids between meals to avoid filling up on these when you are trying to eat.
- Sip fluids little and often rather than large amounts at once, to help prevent vomiting
- Spicy, oily/fried or rich foods may be less well tolerated
- Spend time outside to get fresh air. You may feel better if you don’t get too hot either inside or outside
- Wear comfortable clothes without tight waistbands.

You may want to try and build up your food intake by starting with the following suggestions under each important food group:

**STARCHY CARBOHYDRATES**

- Dry toast, roll, bagel, crumpet, wrap, pitta, chapatti or other types of bread
- Dry crackers, crispbreads, rye crackers, rice cakes, oat cakes, breadsticks
- Jacket or mashed potato
- Cereal, dry or with mil
- Plain rice, pasta, noodles
- Cous cous, bulgar wheat, quinoa and other grains
- Pretzels

**NON DAIRY PROTEIN**

- Grilled or baked plain chicken/turkey breast
- Grilled or baked lean beef or ham
- Ready to eat cold meats
- Plain white/ non oily fish fingers
- Beans and lentils
- Hard boiled or scrambled egg
- Nuts

**DAIRY AND ALTERNATIVES**

- Milk or alternatives mil drinks (nuts, oat, rice, soya)
- Yoghurts
- Custards, rice pudding or other milk puddings
- Plain, hard or pasteurized cheeses.

**FRUITS AND VEGETABLES**

- Raw, cut vegetables such as carrots, cucumber, peppers
- Raw, cut fruits such as apples, pears melon
- Citrus fruits, oranges, satsuma, tangerine, grapefruit
- Tinned fruit
- Saps of fruit juice

**SNACKS THAT CAN GIVE EXTRA ENERGY AND PROTEIN**

- Cereal bar
- Crisps
- Cheese and crackers
- Plain biscuits, ginger biscuits, water biscuits
- Plain sponge caked
- Plain scone
- Hot cross bun or tea cake
- Jelly
- Ice cream, ice lollies, sorbet
- Frozen fruit
- Mousses
- Soups

If you are managing to eat and keep down at least some small meals portions, aim to fortify these by adding small/moderate amounts of high energy ingredients such as butter, grated cheese, yoghurt, cream, sour cream, crème fraiche, milk powder, sugar, syrup, jam and peanut butter, as tolerated.

**GETTING ENOUGH FLUID**

Replacing fluids lost through vomiting is very important to prevent dehydration. Getting enough is difficult and water isn’t always the best option. The following liquids or, methods of taking liquids, may be better tolerated, whilst in some cases also providing extra energy and nutrients.

- Fizzy drinks or sports drinks
- Juices, squash, cordial
- Milk, milkshakes or smoothies
- Ginger ale
- Suck on ice cubes made from water, flat fizzy drinks or squash
- Jelly and ice lollies
- Freeze half a bottle of water or squash and top up with cold water/ extra squash to keep it cold for longer
- Continually sip every small amount of fluid through a straw

**REFERENCES/ FURTHER INFORMATION**

<http://www.pregnancysicknesssupport.org.uk/help/women-suffering/eating-advice/>

<http://www.rcog.org.uk/en/patient/patient-leaflets/pregnancy-sickness/>
