# Supplementary material for: Watermelon and dietary advice compared to dietary advice alone following hospitalization for hyperemesis gravidarum: a randomized controlled trial
Source: BMC Pregnancy Childbirth. 2023 Jun 17;23:450. doi: 10.1186/s12884-023-05771-7 (PMC10276427; doi:10.1186/s12884-023-05771-7)
Supplement: Supplementary file 2 — Additional file 2: Supplementary Material S2. Standard operating protocol: food preparation and storage. [file 12884_2023_5771_MOESM2_ESM.docx]

**STANDARD OPERATING PROTOCOL: FOOD PREPARATION AND STORAGE**

The participants required to wash their hands prior to preparation of the watermelon. Use a clean cut board and knife.

**Preparation and storage of watermelon:**

1. Wash the outer part of watermelon under clean running water prior to preparation
2. Place watermelon on a flat surface in the elongated axis
3. Cut the watermelon into half symmetrically
4. Half of the watermelon cut into further half ( 1/4^th^ of whole watermelon)
5. One quarter of the whole watermelon cut into half ( 1/8^th^ of whole watermelon)
6. 1/8^th^ of the watermelon should be finish eating in a day. Slice it into desired shape and size, to be taken before each meal and snack on as needed.
7. These sliced pieces need to be prepared fresh each time.
8. Do not slice into small pieces and keep in refrigerator.
9. The remaining uncut watermelon must be wrapped with a plastic wrap and stored in the refrigerator (stored at 1-4 degrees Celsius)
10. During each preparation, the exposed portion of the cut watermelon should be re-cut and discarded (approximately 1 cm thickness) first prior to the preparation for the next meal.
11. A whole watermelon can be kept at room temperature for 2 weeks.
12. Refrigerated remaining watermelon after cutting should be used within a week.
13. If the balance watermelon not used within a week, the cut watermelon should be discarded in the usual manner.

**PREPARATION AND STORAGE OF WATERMELON**

1. Place watermelon on a flat surface after cleaning under the running tap water. Cut the watermelon into half


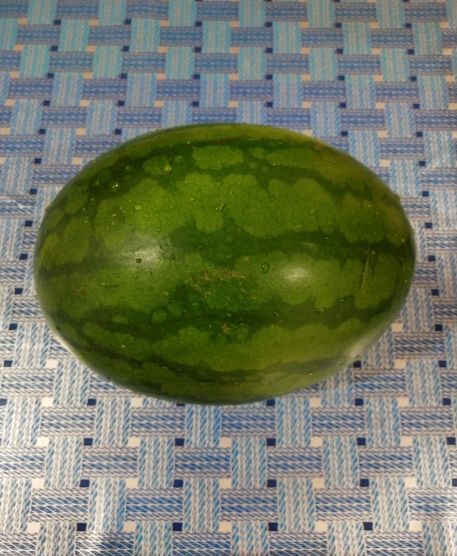

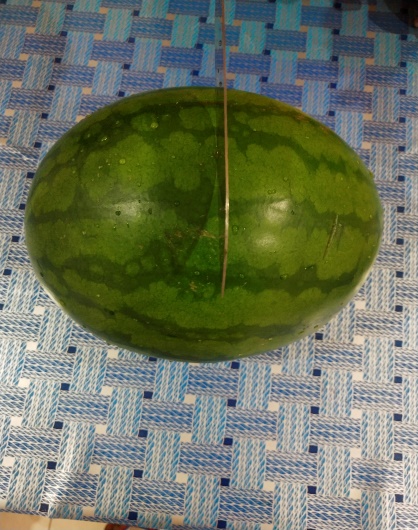

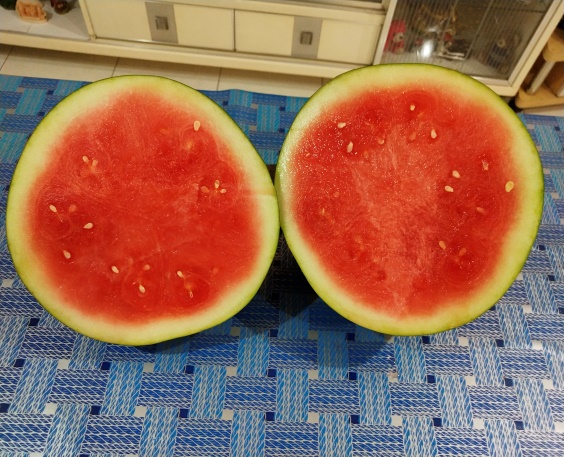


2. One half of the watermelon cut into half ( 1/4 of a whole watermelon)


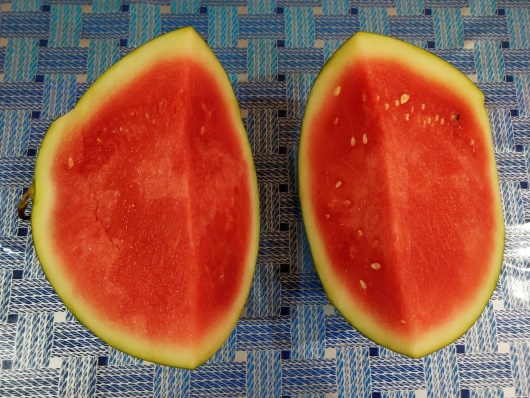

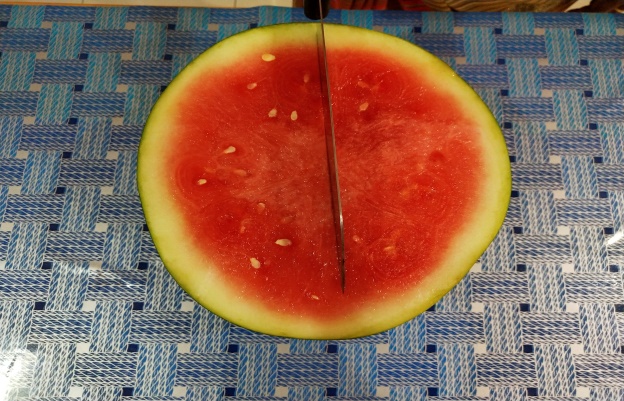


3. One quarter (1/4) of the watermelon cut into further half (1/8 of a whole watermelon)


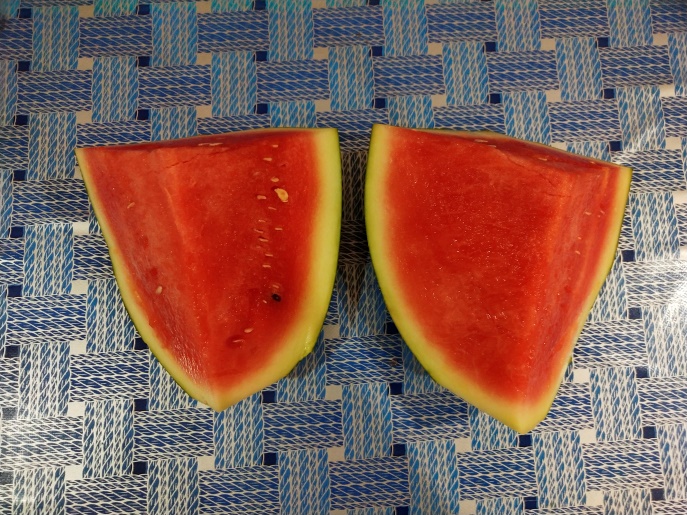

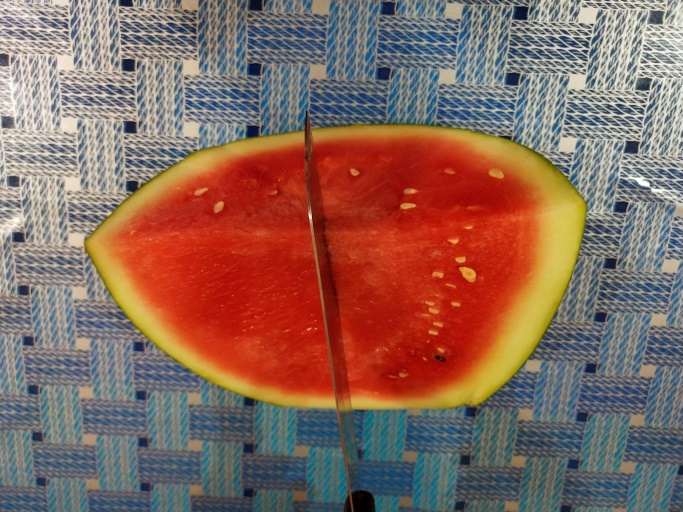


4. Need to eat minimum 1/8^th^ of a whole watermelon in a day.


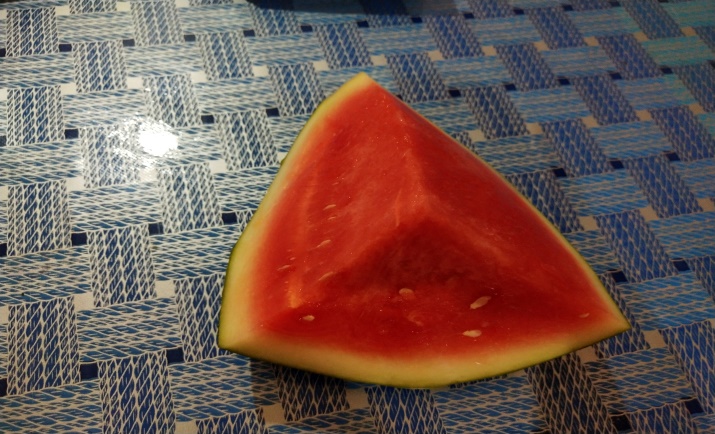

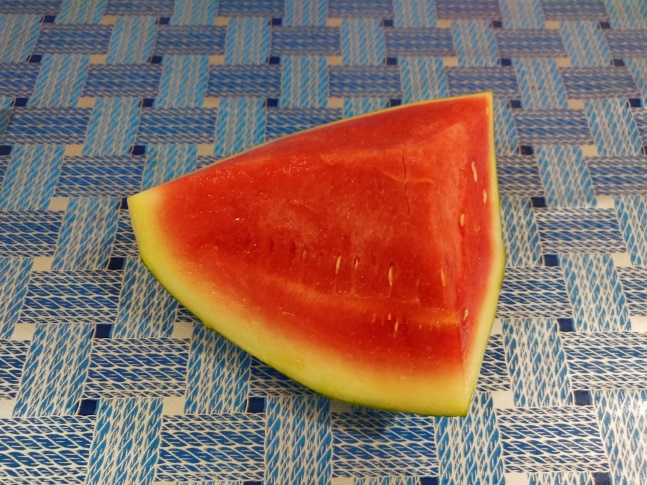


5. The remaining watermelon need to be wrapped with clean food wrapper separately and stored in refrigerator.


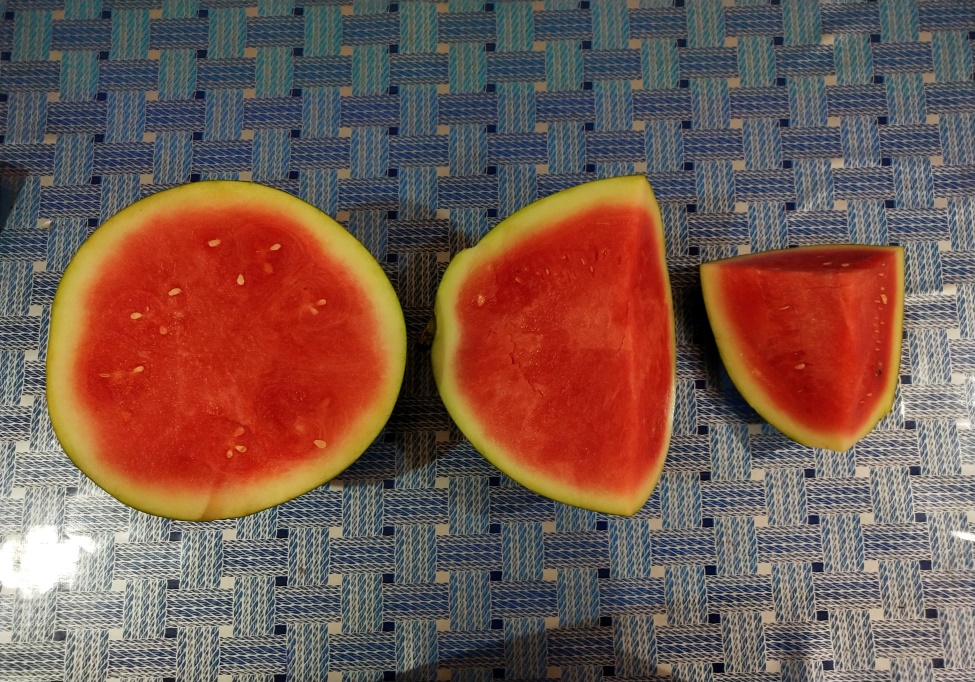


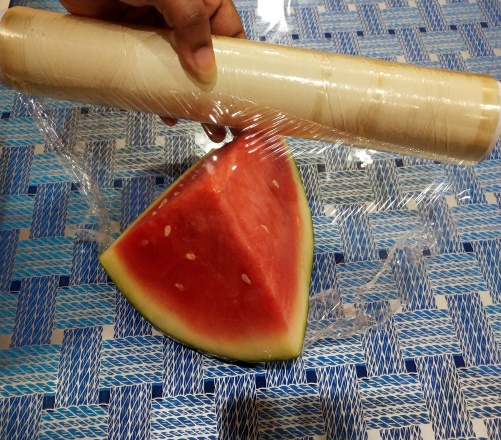

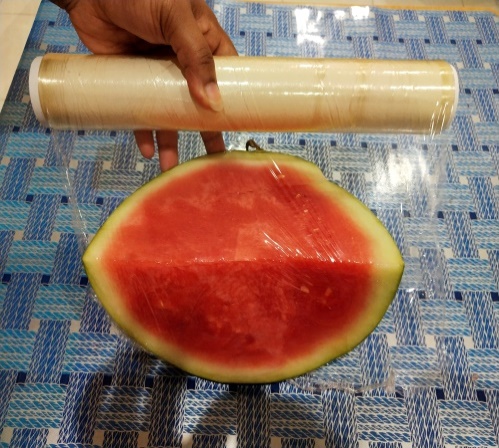


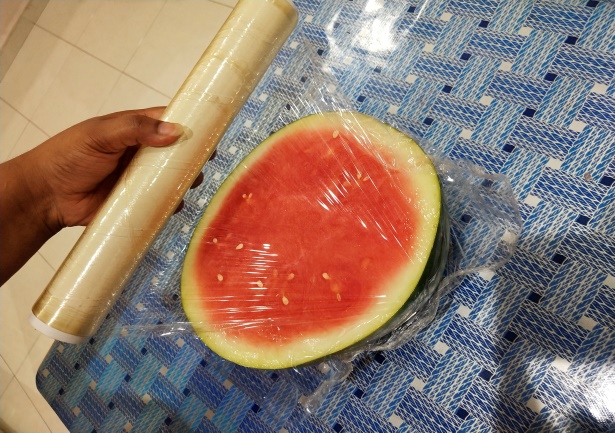


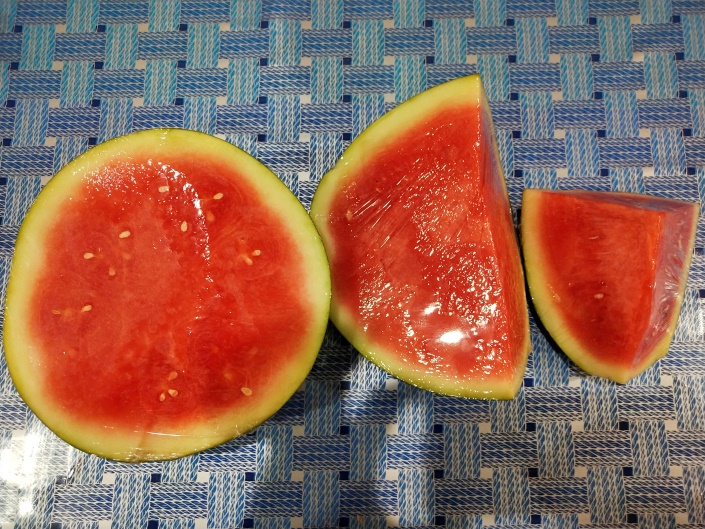


6. Participants need to finish the smallest cut portion first to maintain the freshness of the watermelon.
