# Supplementary material for: Watermelon and dietary advice compared to dietary advice alone following hospitalization for hyperemesis gravidarum: a randomized controlled trial
Source: BMC Pregnancy Childbirth. 2023 Jun 17;23:450. doi: 10.1186/s12884-023-05771-7 (PMC10276427; doi:10.1186/s12884-023-05771-7)
Supplement: Supplementary file 3 — Additional file 3: Supplementary Material S3. Standard operating protocol: body weight measurement. [file 12884_2023_5771_MOESM3_ESM.docx]

STANDARD OPERATING PROTOCOL: BODY WEIGHT MEASUREMENT

1. Place the digital weighing scale in a flat surface.

2. Turn on the scale’s display as a check for the operational function.

3. Wait for the display to show 0.0

4. Ensure shoes, any heavy clothing, any other heavy objects on them, such as things in pockets (mobile phones, wallets, and loose change), belt and any heavy jewellery, chunky watches. Should be in minimal underclothing and single layer thin clothing.

5. Empty the bladder before taking measurement.

6. Measurement should be done in the morning as 1st thing after waking up before drinking water or breakfast.

7. Step onto the scale; stand in the middle of the platform, with head erect and eyes looking straight. They should stand still with weight evenly distributed on both feet, to achieve an accurate reading. The display should then show a fixed weight.

8. Repeat the procedure for 3 times and record

9. All three readings need to be reported to the investigator during interview at 1st and 2nd week after discharge.
